# Complete genome sequence of *Syntrophobotulus glycolicus* type strain (FlGlyR^T^)

**DOI:** 10.4056/sigs.2004684

**Published:** 2011-07-01

**Authors:** Cliff Han, Romano Mwirichia, Olga Chertkov, Brittany Held, Alla Lapidus, Matt Nolan, Susan Lucas, Nancy Hammon, Shweta Deshpande, Jan-Fang Cheng, Roxanne Tapia, Lynne Goodwin, Sam Pitluck, Marcel Huntemann, Konstantinos Liolios, Natalia Ivanova, Ioanna Pagani, Konstantinos Mavromatis, Galina Ovchinikova, Amrita Pati, Amy Chen, Krishna Palaniappan, Miriam Land, Loren Hauser, Evelyne-Marie Brambilla, Manfred Rohde, Stefan Spring, Johannes Sikorski, Markus Göker, Tanja Woyke, James Bristow, Jonathan A. Eisen, Victor Markowitz, Philip Hugenholtz, Nikos C. Kyrpides, Hans-Peter Klenk, John C. Detter

**Affiliations:** 1 DOE Joint Genome Institute, Walnut Creek, California, USA; 2 Los Alamos National Laboratory, Bioscience Division, Los Alamos, New Mexico, USA; 3 Jomo Kenyatta University of Agriculture and Technology, Kenya; 4 Biological Data Management and Technology Center, Lawrence Berkeley National Laboratory, Berkeley, California, USA; 5 Oak Ridge National Laboratory, Oak Ridge, Tennessee, USA; 6 DSMZ - German Collection of Microorganisms and Cell Cultures GmbH, Braunschweig, Germany; 7 HZI – Helmholtz Centre for Infection Research, Braunschweig, Germany; 8 University of California Davis Genome Center, Davis, California, USA; 9 Australian Centre for Ecogenomics, School of Chemistry and Molecular Biosciences, The University of Queensland, Brisbane, Australia

**Keywords:** glycolate-oxidizing, Gram-negative staining with Gram-positive cell wall structure, strictly anaerobic, chemotrophic, mesophilic, non-motile, rod-shaped, spore-forming, *Peptococcaceae*, *Clostridiales*, GEBA

## Abstract

*Syntrophobotulus glycolicus* Friedrich *et al*. 1996 is currently the only member of the genus *Syntrophobotulus* within the family *Peptococcaceae*. The species is of interest because of its isolated phylogenetic location in the genome-sequenced fraction of tree of life. When grown in pure culture with glyoxylate as carbon source the organism utilizes glyoxylate through fermentative oxidation, whereas, when grown in syntrophic co-culture with homoacetogenic or methanogenic bacteria, it is able to oxidize glycolate to carbon dioxide and hydrogen. No other organic or inorganic carbon source is utilized by *S. glycolicus*. The subdivision of the family *Peptococcaceae* into genera does not reflect the natural relationships, particularly regarding the genera most closely related to *Syntrophobotulus*. Both *Desulfotomaculum* and *Pelotomaculum* are paraphyletic assemblages, and the taxonomic classification is in significant conflict with the 16S rRNA data. *S. glycolicus* is already the ninth member of the family *Peptococcaceae* with a completely sequenced and publicly available genome. The 3,406,739 bp long genome with its 3,370 protein-coding and 69 RNA genes is a part of the *** G****enomic* *** E****ncyclopedia of* *** B****acteria and* *** A****rchaea * project.

## Introduction

Strain FlGlyR^T^ (= DSM 8271) is the type strain of *Syntrophobotulus glycolicus* within the monotypic genus *Syntrophobotulus* [1], which is affiliated to the family *Peptococcaceae* within the order *Clostridiales* [2]. The genus name is derived from the latinized Greek *syntrophos* meaning *having grown up with one*, and the Latin *botulus*, sausage, a syntrophic sausage-like item [3]. The species epithet is derived from the Neo-Latin *acidum glycolicum* meaning 'glycolic acid', 'referring to the key substrate of this species, glycolic acid [3]. The major characteristic that differentiates this genus from other bacteria is the ability to oxidize glyoxylate under anaerobic conditions [3]. The major source of glycolate in nature is excretion by algae and other photoautotrophs and chemoautotrophs [4-7]. Strain FlGlyR^T^ was isolated from anoxic sewage sludge in Konstanz, Germany [3], but was also mentioned in earlier reports [8]. No further isolates have been reported until now. Here we present a summary classification and a set of features for *S. glycolicus* FlGlyR^T^, together with the description of the complete genomic sequencing and annotation.

## Classification and features

A representative genomic 16S rRNA sequence of strain FlGlyR^T^ was compared using NCBI BLAST [9] under default settings (*e.g*., considering only the high-scoring segment pairs (HSPs) from the best 250 hits) with the most recent release of the Greengenes database [10] and the relative frequencies of taxa and keywords (reduced to their stem [11]) were determined, weighted by BLAST scores. The most frequently occurring genera were *Desulfitobacterium* (45.4%), *Desulfosporosinus* (19.3%), *Dehalobacter* (18.0%), *Heliobacterium* (13.8%) and *Syntrophobotulus* (2.6%) (85 hits in total). Regarding the single hit to sequences from members of the species, the average identity within HSPs was 99.7%, whereas the average coverage by HSPs was 99.7%. Among all other species, the one yielding the highest score was *Dehalobacter restrictus* (Y10164), which corresponded to an identity of 95.0% and an HSP coverage of 85.2%. (Note that the Greengenes database uses the INSDC (= EMBL/NCBI/DDBJ) annotation, which is not an authoritative source for nomenclature or classification.) The highest-scoring environmental sequence was AJ278164 (‘*Dehalobacter* sp. clone SHD-11' [12]), which showed an identity of 95.3% and an HSP coverage of 86.8%. The most frequently occurring keywords within the labels of environmental samples which yielded hits were 'soil' (6.5%), 'microbi' (6.4%), 'respons' (4.8%), 'paddi, rice' (4.6%) and 'condit' (4.5%) (165 hits in total). The most frequently occurring keyword within the labels of environmental samples which yielded hits of a higher score than the highest scoring species was 'dehalobact' (100.0%) (1 hit in total). The BLAST analysis results concur with earlier reports on the ecology and the physiology of the isolate whereby it was isolated from a co-culture with other sulfate-reducing bacteria [3,8].

**Figure 1:**
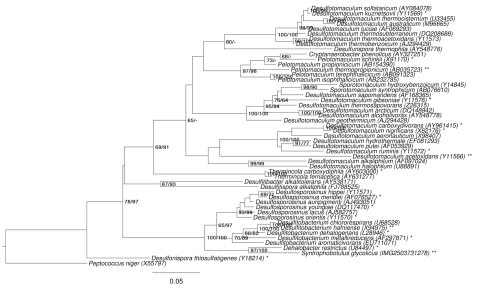
Phylogenetic tree highlighting the position of *S. glycolicus* relative to the type strains of the most closely related genera within the family *Peptococcaceae*. The tree was inferred from 1,306 aligned characters [13,14] of the 16S rRNA gene sequence under the maximum likelihood (ML) criterion [15] and rooted with the type species of the family. The branches are scaled in terms of the expected number of substitutions per site. Numbers adjacent to the branches are support values from 700 ML bootstrap replicates [16] (left) and from 1,000 maximum parsimony bootstrap replicates [17] (right) if larger than 60%. Lineages with type strain genome sequencing projects registered in GOLD [18] are labeled with one asterisk, those also listed as 'Complete and Published' with two asterisks [19-21].

Figure 1 shows the phylogenetic neighborhood of *S. glycolicus* in a 16S rRNA based tree. The sequences of the four 16S rRNA gene copies in the genome differ from each other by up to eight nucleotides, and differ by up to 14 nucleotides from the previously published 16S rRNA sequence X99706, which contains two ambiguous base calls.

As two of the genera selected for Figure 1, *Desulfotomaculum* and *Pelotomaculum*, appeared as paraphyletic in the tree, we conducted both unconstrained heuristic searches for the best tree under the maximum likelihood (ML) [15] and maximum parsimony (MP) criterion [17] as well as searches constrained for the monophyly of all genera (for details of the data matrix see the figure caption). The best-known ML tree had a log likelihood of -12,054.61, whereas the best trees found under the constraint had a log likelihood of -12,209.39 and were significantly worse in the SH test as implemented in RAxML [15] (p < 0.01). The best-known MP trees had a score of 2,018 whereas the best trees found under the constraint had a score of 2,076 and were significantly worse in the KH test as implemented in PAUP* [17] (p < 0.0001). Accordingly, the current classification of the group is in significant conflict with the 16S rRNA data and apparently does not reflect its natural relationships. The classification could be improved if combinations of phenotypic character states were found which characterize a set of appropriately rearranged, then monophyletic genera. However, it might also be that the goal to 'define' each genus in terms of unique combinations of few, potentially arbitrarily selected character states is over-ambitious, if not misleading in this group of organisms. Apparently a taxonomic revision of the family appears to be necessary which focuses more strongly on the genealogy of the organisms than previous treatments.

**Figure 2:**
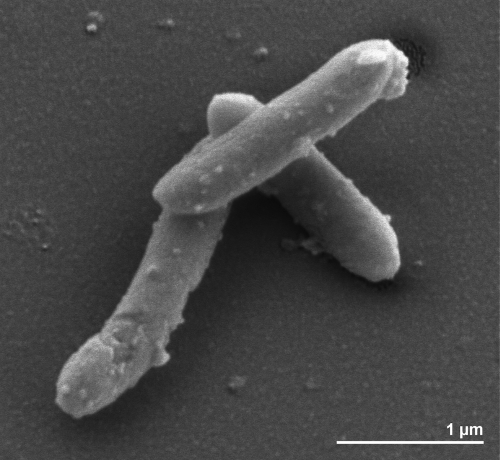
Scanning electron micrograph of *S. glycolicus* FlGlyR^T^

Cells of strain FlGlyR^T^ are Gram-positive, spore forming and slightly curved rods of 2.5-3.5 by 0.5 µm in size [3] (Figure 2). Though the organism is reported to be non-motile, numerous genes associated with flagellar motility are present in the genome (see below). Growth occurs between 15°C and 37°C with an optimum at 28°C, and in a pH range of 6.7 to 8.3, with an optimum at pH 7.3 [3] (Table 1). The reported habitat for this strain is sewage sludge and anoxic freshwater sediments [3]. Initial isolation condition was from defined co-cultures of fermenting bacteria with homoacetogenic or methanogenic bacteria which converted glycolate completely to CO_2_ and H_2_, with concomitant reduction of CO_2_ to either acetate or methane [3,8]. Later strain FlGlyR^T^ was identified as the primary fermenting partner in these co-cultures and glyoxylate was the substrate [3]. Strain FlGlyR^T^ grows optimally in freshwater medium although growth also occurred in brackish-water medium with 110 mM NaC1 and 5 mM MgCl [3]. Strain FlGlyR^T^ is strictly anaerobic, growing chemotrophically in pure culture by fermentative oxidation of glyoxylate [3]. In pure culture, glyoxylic acid is fermented to carbon dioxide, hydrogen, and glycolic acid [3]. However, in syntrophic co-culture with, e.g., *Methanospirillum hungatei* or *Acetobacterium woodii* as a partner, glycolic acid is converted to carbon dioxide and hydrogen [3]. Glycolate oxidation to glyoxylate and vice versa is coupled to a membrane-bound electron transport system that catalyzes either a proton potential-driven reversed electron transport from glycolate to hydrogen or a hydrogen-dependent glyoxylate reduction coupled to ATP synthesis by electron transport phosphorylation [34,35]. Due to the oxygenase activity of the D-ribulose-1,5-bisphosphate carboxylase at low CO_2_ and high O_2_ concentrations, the phosphoglycolate formed in these organisms is subsequently dephosphorylated to glycolate [8]. It is reported that no other organic or inorganic substrates are used [3], even though a total of 78 carbohydrate transport and metabolism genes are found the genome of this organism (COGS table). Neither sulfate, sulfite, thiosulfate, elemental sulfur, nor nitrate are reduced [3].

**Table 1:** Classification and general features of *S. glycolicus* FlGlyR^T^ according to the MIGS recommendations [22] and the NamesforLife database [23].

**MIGS ID**	**Property**	**Term**	**Evidence code**
	Current classification	Domain *Bacteria*	TAS [24]
		Phylum *Firmicutes*	TAS [25,26]
		Class *Clostridia*	TAS [27,28]
		Order *Clostridiales*	TAS [29,30]
		Family *Peptococcaceae*	TAS [29,31]
		Genus *Syntrophobotulus*	TAS [3]
		Species *Syntrophobotulus glycolicus*	TAS [3]
		Type strain FlGlyR	TAS [3]
	Gram stain	negative	TAS [3]
	Cell shape	rod shaped, slightly curved	TAS [3]
	Motility	non-motile	TAS [3]
	Sporulation	sporulating	TAS [3]
	Temperature range	15°C-37°C	TAS [3]
	Optimum temperature	28°C	TAS [3]
	Salinity	tolerates ~6% NaCl	TAS [3]
MIGS-22	Oxygen requirement	strictly anaerobic	TAS [3]
	Carbon source	glyoxylate	TAS [3]
	Energy metabolism	chemotrophic	TAS [3]
MIGS-6	Habitat	marine, sludge, fresh water	TAS [3]
MIGS-15	Biotic relationship	free-living	TAS [3]
MIGS-14	Pathogenicity	not reported	
	Biosafety level	1	TAS [32]
	Isolation	anoxic sludge from municipal sewage treatment plant	TAS [3,8]
MIGS-4	Geographic location	Konstanz, Germany	TAS [3,8]
MIGS-5	Sample collection time	1991 or before	TAS [3,8]
MIGS-4.1	Latitude	47.67	NAS
MIGS-4.2	Longitude	9.16	NAS
MIGS-4.3	Depth	unknown	
MIGS-4.4	Altitude	about 420 m	NAS

Evidence codes - IDA: Inferred from Direct Assay (first time in publication); TAS: Traceable Author Statement (i.e., a direct report exists in the literature); NAS: Non-traceable Author Statement (i.e., not directly observed for the living, isolated sample, but based on a generally accepted property for the species, or anecdotal evidence). These evidence codes are from of the Gene Ontology project [33]. If the evidence code is IDA, the property was directly observed by one of the authors or an expert mentioned in the acknowledgements.

### Chemotaxonomy

Strain FlGlyR^T^ has no cytochromes and the cells contain menaquinone-7-10, with MK-9 as major fraction [3]. Although the cells stain Gram-negative, the ultrastructural analysis shows a Gram-positive cell wall architecture [3].

## Genome sequencing and annotation

### Genome project history

This organism was selected for sequencing on the basis of its phylogenetic position [36], and is part of the *** G****enomic* *** E****ncyclopedia of* *** B****acteria and* *** A****rchaea * project [37]. The genome project is deposited in the Genome On Line Database [18] and the complete genome sequence is deposited in GenBank. Sequencing, finishing and annotation were performed by the DOE Joint Genome Institute (JGI). A summary of the project information is shown in Table 2.

**Table 2:** Genome sequencing project information

**MIGS ID**	**Property**	**Term**
MIGS-31	Finishing quality	Finished
MIGS-28	Libraries used	Three genomic libraries: one 454 pyrosequence standard library, one 454 PE library (13 kb insert size), one Illumina library
MIGS-29	Sequencing platforms	Illumina GAii, 454 GS FLX Titanium
MIGS-31.2	Sequencing coverage	167.0 × Illumina; 48.0 × pyrosequence
MIGS-30	Assemblers	Newbler version 2.3 (Roche), Velvet 0.7.63, phrap SPS - 4.24
MIGS-32	Gene calling method	Prodigal 1.4, GenePRIMP
	INSDC ID	CP002547
	Genbank Date of Release	March 4, 2011
	GOLD ID	Gc01670
	NCBI project ID	38111
	Database: IMG-GEBA	2503707006
MIGS-13	Source material identifier	DSM 8271
	Project relevance	Tree of Life, GEBA

### Growth conditions and DNA isolation

*S. glycolicus* FlGlyR^T^, DSM 8271, was grown anaerobically in DSMZ medium 298b (FlGlyM-medium) [38] at 28°C. DNA was isolated from 0.5-1 g of cell paste using Jetflex Genomic DNA Purification kit (GENOMED 600100) following the standard protocol as recommended by the manufacturer, adding 10 µL proteinase K to the standard lysis solution for 50 minutes at 58°C. DNA is available through the DNA Bank Network [39].

### Genome sequencing and assembly

The genome was sequenced using a combination of Illumina and 454 sequencing platforms. All general aspects of library construction and sequencing can be found at the JGI website [40]. Pyrosequencing reads were assembled using the Newbler assembler. The initial Newbler assembly consisting of 38 contigs in two scaffolds was converted into a phrap [41] assembly by making fake reads from the consensus, to collect the read pairs in the 454 paired end library. Illumina sequencing data (602.6 Mb) was assembled with Velvet [42] and the consensus sequences were shredded into 1.5 kb overlapped fake reads and assembled together with the 454 data. 454 draft assembly was based on 163.5 Mb 454 draft data and all of the 454 paired end data. Newbler parameters are -consed -a 50 -l 350 -g -m -ml 20. The Phred/Phrap/Consed software package [41] was used for sequence assembly and quality assessment in the subsequent finishing process. After the shotgun stage, reads were assembled with parallel phrap (High Performance Software, LLC). Possible mis-assemblies were corrected with gapResolution [40], Dupfinisher, or sequencing cloned bridging PCR fragments with subcloning or transposon bombing (Epicentre Biotechnologies, Madison, WI) [43]. Gaps between contigs were closed by editing in Consed, by PCR and by Bubble PCR primer walks (J.-F. Chang, unpublished). A total of 331 additional reactions were necessary to close gaps and to raise the quality of the finished sequence. Illumina reads were also used to correct potential base errors and increase consensus quality using a software Polisher developed at JGI [44]. The error rate of the completed genome sequence is less than 1 in 100,000. Together, the combination of the Illumina and 454 sequencing platforms provided 215.0 × coverage of the genome. The final assembly contained 327,738 pyrosequence and 15,336,223 Illumina reads.

### Genome annotation

Genes were identified using Prodigal [45] as part of the Oak Ridge National Laboratory genome annotation pipeline, followed by a round of manual curation using the JGI GenePRIMP pipeline [46]. The predicted CDSs were translated and used to search the National Center for Biotechnology Information (NCBI) non-redundant database, UniProt, TIGR-Fam, Pfam, PRIAM, KEGG, COG, and InterPro databases. Additional gene prediction analysis and functional annotation was performed within the Integrated Microbial Genomes - Expert Review (IMG-ER) platform [47].

## Genome properties

**Figure 3:**
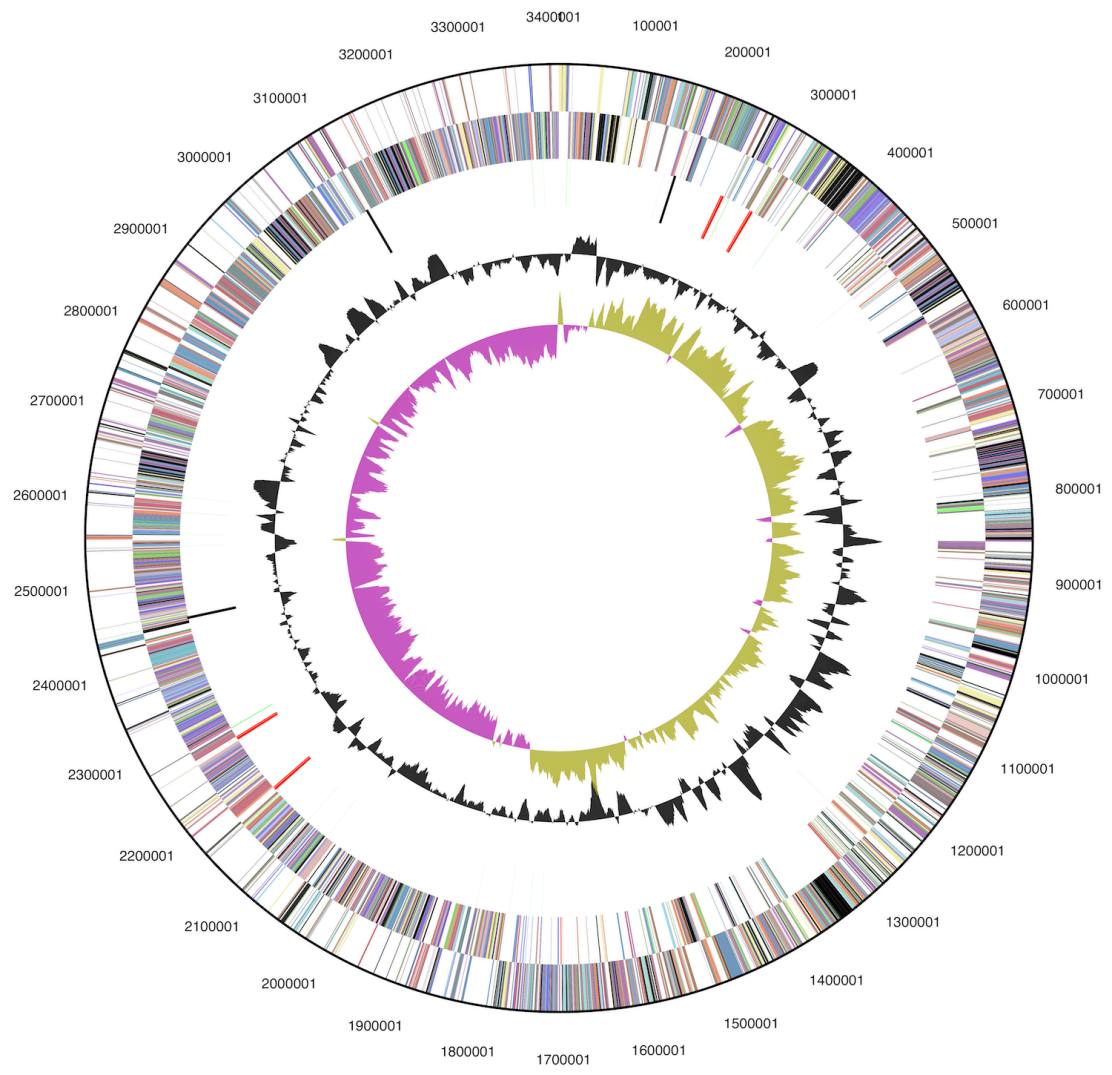
Graphical circular map of chromosome. From outside to the center: Genes on forward strand (color by COG categories), Genes on reverse strand (color by COG categories), RNA genes (tRNAs green, rRNAs red, other RNAs black), GC content, GC skew.

The genome consists of a 3,406,739bp long chromosome with a GC content of 46.4% (Table 3 and Figure 3). Of the 3,439 genes predicted, 3,370 were protein-coding genes, and 69 RNAs; 119 pseudogenes were also identified. The majority of the protein-coding genes (68.7%) were assigned a putative function while the remaining ones were annotated as hypothetical proteins. The distribution of genes into COGs functional categories is presented in Table 4.

**Table 3:** Genome Statistics

**Attribute**	**Value**	**% of Total**
Genome size (bp)	3,406,739	100.00%
DNA coding region (bp)	2,989,609	87.76%
DNA G+C content (bp)	1,579,030	46.35%
Number of replicons	1	
Extrachromosomal elements	0	
Total genes	3,439	100.00%
RNA genes	69	2.01%
rRNA operons	4	
Protein-coding genes	3,370	97.99%
Pseudo genes	119	3.46%
Genes with function prediction	2364	68.74%
Genes in paralog clusters	710	20.65%
Genes assigned to COGs	2,399	69.76%
Genes assigned Pfam domains	2,561	74.47%
Genes with signal peptides	463	13.46%
Genes with transmembrane helices	848	24.66%
CRISPR repeats	2	

**Table 4:** Number of genes associated with the general COG functional categories

**Code**	**value**	**%age**	**Description**
J	152	5.8	Translation, ribosomal structure and biogenesis
A	0	0.0	RNA processing and modification
K	230	8.8	Transcription
L	156	6.0	Replication, recombination and repair
B	1	0.0	Chromatin structure and dynamics
D	33	1.3	Cell cycle control, cell division, chromosome partitioning
Y	0	0.0	Nuclear structure
V	98	3.8	Defense mechanisms
T	162	6.2	Signal transduction mechanisms
M	170	6.5	Cell wall/membrane/envelope biogenesis
N	68	2.6	Cell motility
Z	0	0.0	Cytoskeleton
W	0	0.0	Extracellular structures
U	43	1.7	Intracellular trafficking, secretion, and vesicular transport
O	74	2.8	Posttranslational modification, protein turnover, chaperones
C	156	6.0	Energy production and conversion
G	78	3.0	Carbohydrate transport and metabolism
E	214	8.2	Amino acid transport and metabolism
F	65	2.5	Nucleotide transport and metabolism
H	140	5.4	Coenzyme transport and metabolism
I	49	1.9	Lipid transport and metabolism
P	195	7.5	Inorganic ion transport and metabolism
Q	27	1.0	Secondary metabolites biosynthesis, transport and catabolism
R	282	10.8	General function prediction only
S	220	8.4	Function unknown
-	1,040	30.2	Not in COGs
